# MiR-29c reduces the cisplatin resistance of non-small cell lung cancer cells by negatively regulating the PI3K/Akt pathway

**DOI:** 10.1038/s41598-018-26381-w

**Published:** 2018-05-22

**Authors:** Dian-min Sun, Bu-fu Tang, Zhen-xiang Li, Hong-bo Guo, Jin-ling Cheng, Ping-ping Song, Xin Zhao

**Affiliations:** 1 Department of Thoracic Surgery, Shandong Cancer Hospital affiliated to Shandong University, Shandong Academy of Medical Sciences, Jinan, China; 2 Department of Oncology, the First Affiliated Hospital of Dalian Medical University, Dalian, Liaoning Province, Dalian Shi, China; 3 Department of Radiation Oncology, Shandong Cancer Hospital Affiliated to Shandong University, Shandong Academy of Medical Sciences, Jinan, China; 4 Department of Internal Medicine, Shandong Provincial Hospital affiliated to Shandong University, Jinan, China

## Abstract

In previous studies, miR-29s showed tumor suppressor properties against lung cancer, which improved the survival of patients upon the administration of chemotherapy via an unknown mechanism. Here, we investigated the regulatory effects of miR-29s on the cisplatin resistance of NSCLC cells. The expression of miR-29s was assessed in 130 clinical patients and in cisplatin-treated NSCLS cell lines. MiR-29c expression was decreased in 77% of NSCLC patients. Cisplatin treatment increased the expression of miR-29c and decreased the expression of its oncogenic target AKT2 in NSCLC cell lines. A Kaplan–Meier survival analysis indicated that higher miR-29c levels led to a longer disease-free survival. In particular, patients who experienced cancer recurrences after cisplatin chemotherapy exhibited a lower level of miR-29c expression, suggesting that miR-29c activation may contribute to the chemotherapeutic efficiency of cisplatin. The enforced expression of miR-29c enhanced the cisplatin sensitivity of NSCLC cells, while the knocking down of miR-29c led to cisplatin resistance. MiR-29c amplified the therapeutic effects of cisplatin *in vivo*. Rescue experiments suggested that miR-29c regulates the cisplatin resistance of NSCLS cells by negatively regulating the PI3K/Akt pathway. Overall, our results demonstrated that miR-29c enhances the sensitivity of NSCLC cells to cisplatin by targeting the PI3K/Akt pathway.

## Introduction

Non-small cell lung cancer (NSCLC) is the most common type of lung cancer and is the leading cause of cancer-related mortality^1^. During cancer genesis and development, gene alterations play important functions. Recently, many oncogenes and tumor suppressor genes, both coding and non-coding, have been identified that affect the progression of lung cancer.

MicroRNAs (miRNAs) are a class of small non-coding genes involved in the regulation of the translation and stability of mRNA^2,3^. With respect to cancer, miRNAs have been demonstrated to contribute to cancer initiation and progression by acting as oncogenes or tumor suppressor genes^4–6^. Interestingly, based on the disparate cancer cell type backgrounds, some miRNAs can function as both oncogenes or tumor suppressor genes, such as miR-96^7,8^, miR-217^9–11^, and the miR-29 family (miR-29s).

Some studies have shown that overexpression of miR-29s leads to an epithelial-to-mesenchymal transition as well as exacerbated metastasis that accelerates cancer progression^12,13^. However, studies have also illustrated the different properties of miR-29s. For example, miR-29s could inhibit the growth of tumors in immunodeficient mice by negatively regulating Bcl-2 and Mcl-1 in liver cancer cells^14^. In addition, miR-29s act as tumor suppressors in lung, cervical and gastric cancers, repressing tumor proliferation and metastasis^15–18^. The function of miR-29s in NSCLC was elucidated by D-W Wu *et al*. who showed that miR-29b, which was down regulated by c-Myc, has a tumor suppressor role by targeting FHIT^19^. Importantly, the expression of miR-29b, a miR-29s member, was negatively correlated to tumor aggressiveness and poor outcome of NSCLC, demonstrating a crucial role for miR-29b in NSCLC^19^.

Because of these reports, we were surprised to observe, in the present study, improved outcomes in NSCLC patients treated using chemotherapy, with a high expression of miR-29c, indicating that miR-29c might benefit the chemotherapy. Because of the demonstrated relationship between miRNA expression and chemoresistance of cancers^20–23^, we hypothesized that there could be an association of miR-29c expression with the sensitivity of lung cancer cells to cisplatin.

In the present study, we provide insights into the association of miR-29c expression with cisplatin resistance in NSCLC patients. We have determined that miR-29c was downregulated in NSCLC and report for the first time that overexpression of miR-29c can reduce cisplatin cell resistance *in vitro* and *in vivo*. Our results may contribute to understanding the role miR-29c plays in cisplatin chemoresistance and provide a basis for the exploitation of novel combinations of targeted agents against NSCLC.

## Results

### MiR-29s were downregulated in NSCLC tissue samples

**Figure 1:**
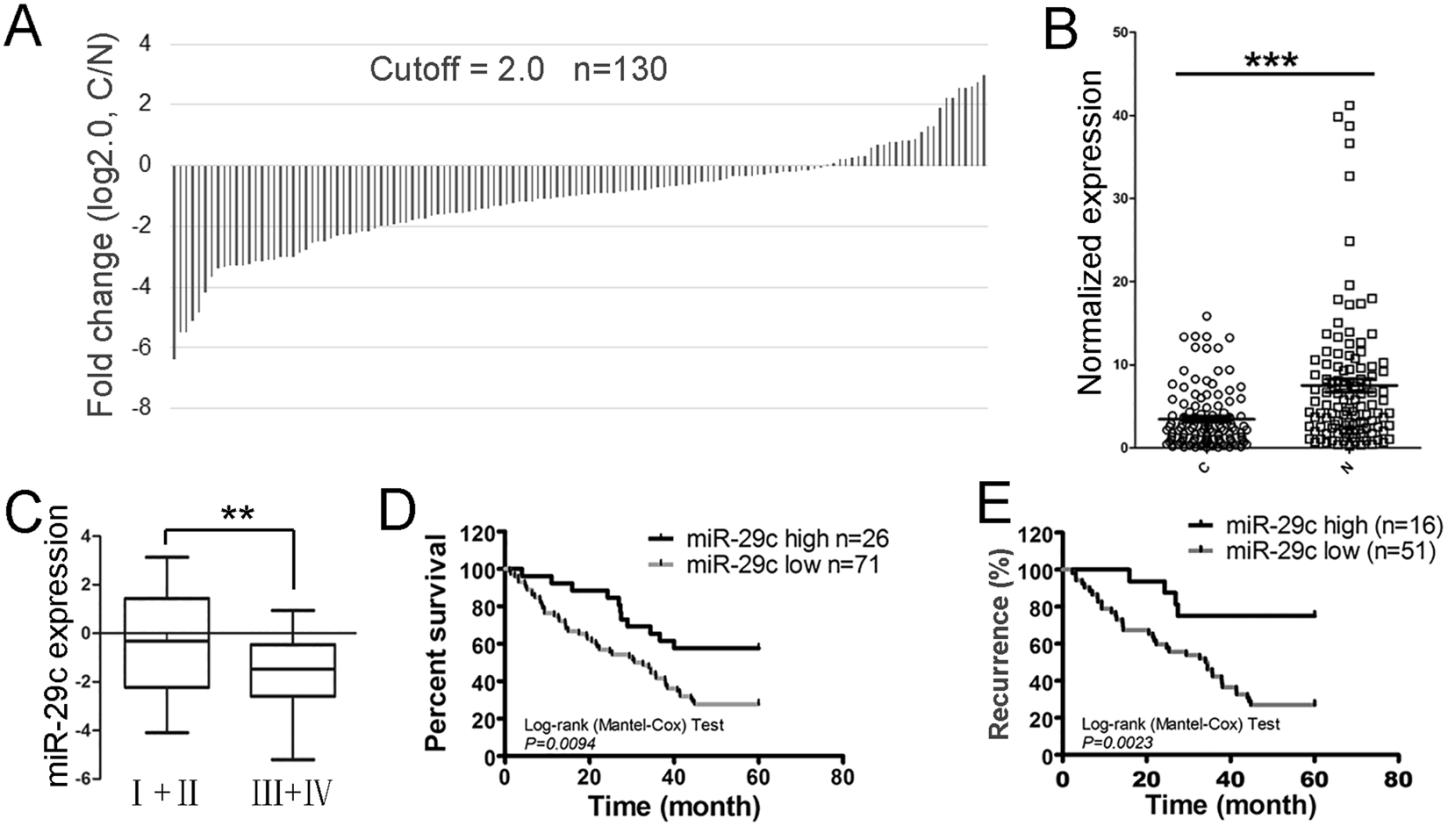
Lowered expression of miR-29c in NSCLC tissues is associated with NSCLC survival and cisplatin resistance. (**A**,**B**) The expression of miR-29c in the cancer tissues was lower than in the adjacent normal tissues examined by using qRT-PCR (*P* < 0.001, paired t-test, two-tailed). MiR-29c expression was calculated by 2^−ΔΔCT^ method and U6 snRNA was used as an endogenous control. C: lung cancer tissues; N: the matched normal lung tissues. (**C**) The relative level of miR-29c in the early pTNM stage (I + II) and advanced pTNM stage (III + IV) patients analyzed by qRT-PCR. (**D**) The Kaplan-Meier survival curve. The relation between the overall survival rate of cancer patients and the miR-29c level was analyzed. Studies were performed with 97 out of 130 patients with NSCLC undergoing chemotherapy after resection and the survival information of whom could be obtained. (**E**) The recurrence of NSCLC patients who underwent cisplatin chemotherapy. NSCLC in patients with low levels of miR-29c tended to recur post cisplatin chemotherapy.

To study the association of miR-29s with the occurrence of NSCLC, we assessed the expression of miR-29s in 130 clinical patients by qRT-PCR. Out of 130 NSCLS samples, the expression of miR-29c was downregulated in 100 cases (77%) compared with noncancerous adjacent tissues using a 1.5-fold expression difference cut-off (Fig. 1A,B). Furthermore, miR-29a/b also showed a downregulation trend, but no significant difference was found between noncancerous adjacent tissues and NSCLC tissues (data not shown). In general, the expression of miR-29c in NSCLC tissues was significantly lower than in adjacent tissues. We further investigated the relative levels of miR-29c between early pTNM stage (I+II) and advanced pTNM stage (III+IV) tumors and found that miR-29c was significantly downregulated in advanced pTNM stage patients (Fig. 1C).

### Low miR-29c expression is associated with NSCLC survival and cisplatin resistance

After demonstrating the decreased expression of miR-29c in NSCLC tissues, we next investigated the correlation between miR-29c and NSCLC survival. Using a Kaplan–Meier survival analysis, we demonstrated that the patients with higher miR-29c levels typically exhibited longer disease-free survival (Fig. 1D). Interestingly, when we investigated the patients with NSCLC undergoing chemotherapy after resection, we found that the cisplatin chemotherapy held better clinical promise for patients with NSCLC exhibiting a higher miR-29c expression in tumors. The NSCLC patients who had lower miR-29c level often recurred after cisplatin treatment (Fig. 1E). This result suggested that miR-29c might be involved in cisplatin resistance regulation in NSCLC cells.

### Cisplatin induces miR-29c expression in SPC-A-1 and A549 cells

**Figure 2:**
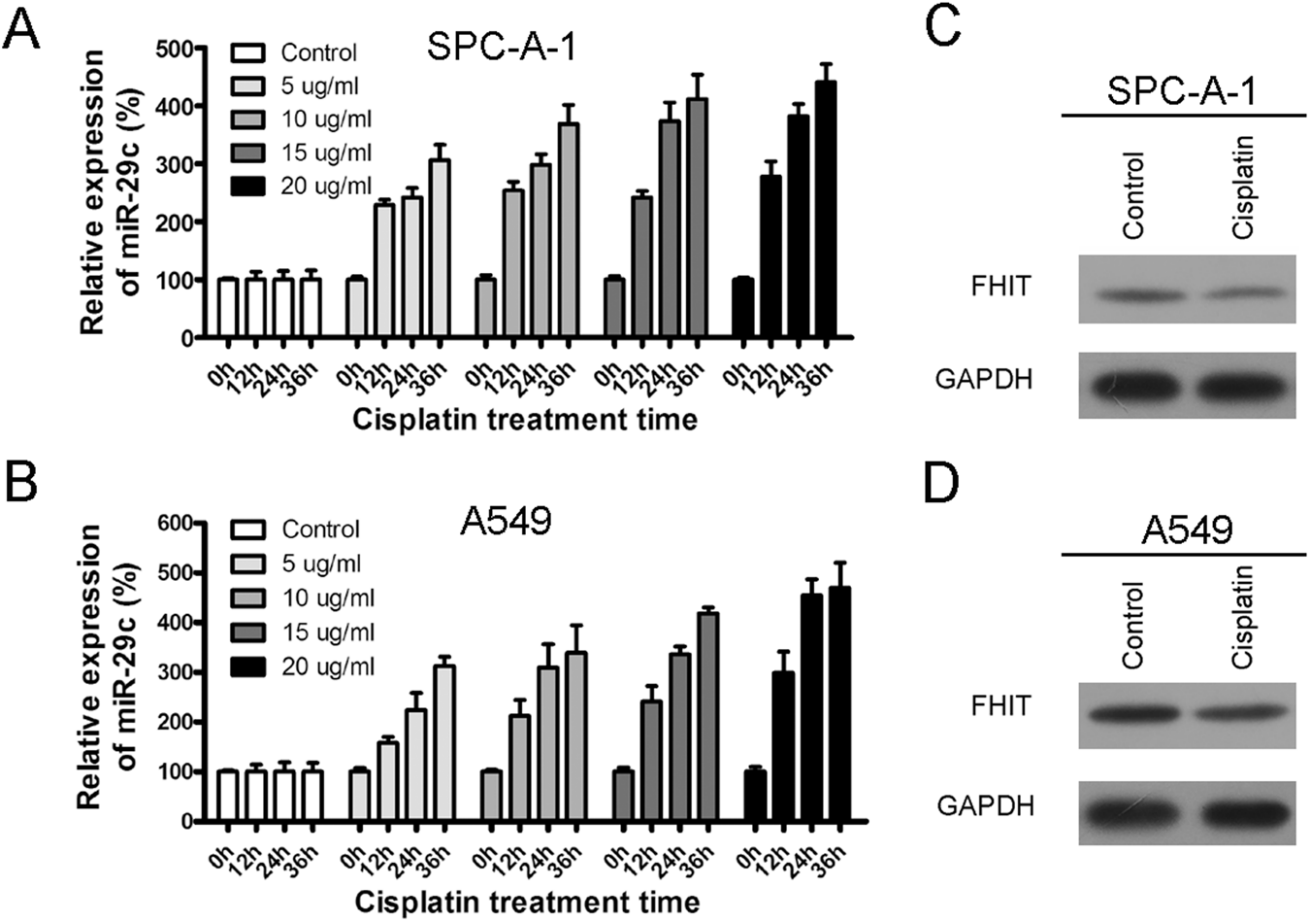
Cisplatin upregulated the expression of miR-29c in NSCLC cells. (**A**,**B**) Either 5, 10, 15, or 20 µg/ml cisplatin was used to treat SPC-A-1 and A549 cells for 0, 12, 24, and 36 hours. The expression of miR-29c was detected by qRT-PCR while the U6 snRNA was used as an endogenous control. (**C**,**D**) A previously demonstrated miR-29c target gene FHIT was downregulated in the protein level with 5 µg/ml cisplatin treatment for 24 hours in SPC-A-1 and A549 cells.

We assessed the expression of miR-29c in SPC-A-1 and A549 cells after a 5, 10, 15, 20 µg/ml cisplatin treatment for 0, 12, 24, and 36 hours. We found that miR-29c expression was significantly upregulated by cisplatin treatment. Importantly, the level of upregulation was positively correlated to the cisplatin concentration and treatment time (Fig. 2A,B). FHIT is a previously demonstrated miR-29c target gene^19^. Here we showed that FHIT was downregulated in the protein level when treated with 5 µg/ml cisplatin for 24 hours in SPC-A-1 and A549 cells. These results demonstrated that cisplatin enhanced miR-29c expression associating with target gene suppression in SPC-A-1 and A549 cells.

### MiR-29c enhances cisplatin sensitivity of lung cancer cells

**Figure 3:**
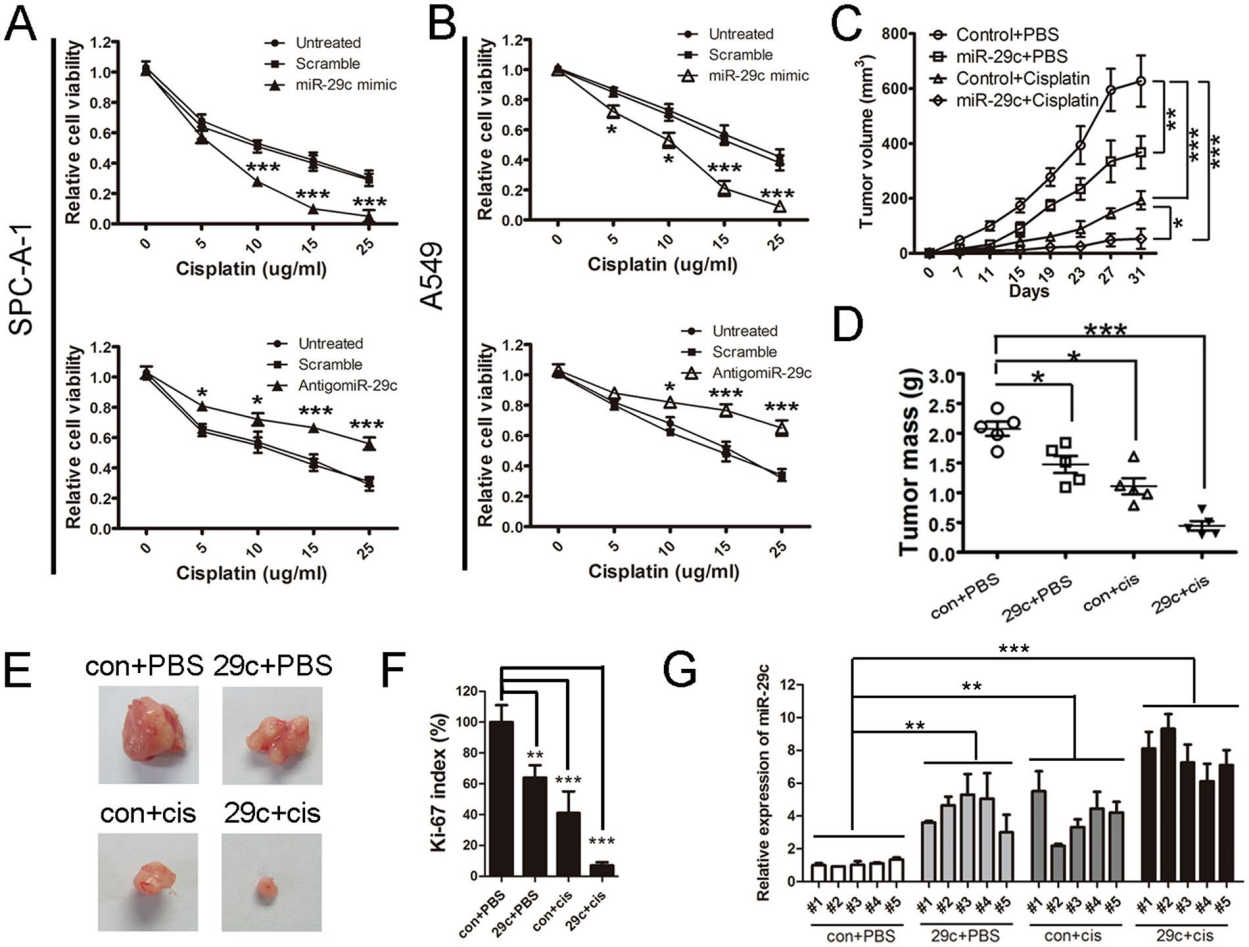
MiR-29c amplified the therapeutic effects of cisplatin *in vitro* and *in vivo*. (**A**,**B**) miR-29c overexpression and knock down showed opposite effects on cisplatin sensitivity in SPC-A-1 and A549 cells. (**C**,**D**) A549 cells expressing miR-29c or a negative control were subcutaneously transplanted in mice. Mice were treated with cisplatin or saline. Tumor volume (**C**) and tumor weight (**D**) were measured. (**E**) Representative photos of tumors in each group. (**F**) Relative Ki-67 index in each group. Data was obtained from 5 mice in each group. Data was normalized to the lowest Ki-67 rate of mice in group “con+PBS”, which was set as 100%. (**G**) The expression of miR-29c in mice tumors. Data was normalized to miR-29c expression of mice #1 in group “con+PBS”. “con” represents mimic control; “cis” represents “cisplatin”; “29c” represents “mimic miR-29c”.

We further verified the association between miR-29c expression levels and cisplatin resistance in lung cancer cells by gain of function and loss of function analyses. The miR-29c was successfully overexpressed or knocked down in SPC-A-1 and A549 cells (Fig. S1A,B). As a result, miR-29c overexpression significantly increased the cisplatin sensitivity of SPC-A-1 and A549 cells. However, when we used antagomiR-29c to knock down miR-29 expression, the cisplatin resistance of miR-29c was significantly increased in SPC-A-1 and A549 cells (Fig. 3A,B). We further investigated this result in nude mice and demonstrated that miR-29c not only inhibited the xenograft tumor growth itself but also amplified the therapeutic effects of cisplatin in *vivo* (Fig. 3C–E). Both miR-29c overexpression and cisplatin treatment induce a decrease of ki-67 index and AKT2 expression in xenograft tumor (Figs 3F and S2A,B). Co-treatment of cancer cell with miR-29c and cisplatin showed the strongest inhibition, indicating that miR-29c amplified the proliferation inhibition effect of cisplatin in lung cancer cell. Besides the immunohistochemical staining, Real-time PCR demonstrated the same results in AKT2 mRNA level in mice tumors (Fig. S2C). While miR-29c expression showed totally opposite tendency to AKT2 expression (Fig. 3G).

### MiR-29c enhances lung cancer cell cisplatin sensitivity by targeting AKT2

**Figure 4:**
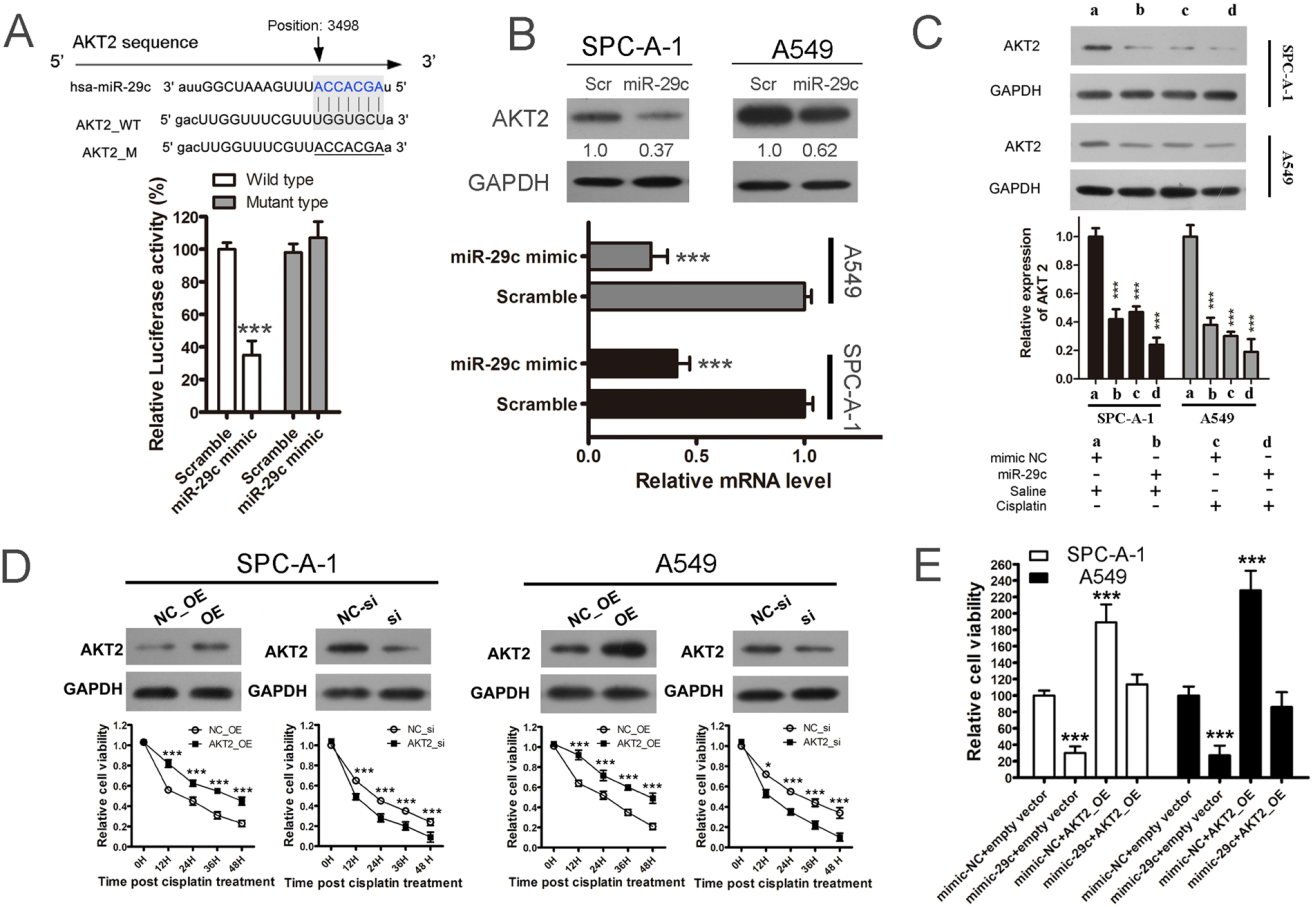
MiR-29c enhances cisplatin sensitivity of lung cancer cells by repressing AKT2 expression. (**A**) AKT2 is a potential target gene of miR-29c. Schematic representation of AKT2 3′-UTRs showing putative miR-29c target site (up). Luciferase activity assay with wild-type AKT2 3′-UTRs constructs and mutated luciferase constructs (down). (**B**) Cisplatin downregulated the expression of AKT2 at both the protein and mRNA levels in SPC-A-1 and A549 cell. Scr/Scramble: microRNA mimic negative control. MiR-29c: miR-29c mimic. (**C**) Both cisplatin treatment and miR-29c overexpression reduce the expression of AKT2. We used 10 µg/ml of cisplatin to treat SPC-A-1 and A549 cells for 12 hours and the AKT2 protein level and RNA level was detected by western blot or real-time PCR respectively. Mimic NC: microRNA mimic negative control. (**D**) Overexpression of AKT2 led to higher cell viability while knocking down of AKT2 led to lower cell viability post cisplatin treatment. AKT2 was overexpressed or knocked down by pcDNA3.1 vector or siRNAs transfection. NC-OE: pcDNA3.1 empty vector used for overexpression negative control. OE: AKT2 overexpression. NC-si: siRNA negative control. Si: siRNA used for AKT2 knockdown. (**E**) Gene rescue experiments demonstrated that miR-29c could enhance the cisplatin sensitivity by negatively regulating AKT2. Mimic-NC: microRNA mimic negative control. AKT_OE: AKT2 gene overexpression by pcDNA3.1 system.

Previous research demonstrated that miR-29c can target and negatively regulate the expression of the oncogene AKT2 and thus plays a role as a tumor suppressor gene in gastric cancer^24^. To test whether it can also target AKT2 in lung cancer, we first performed dual luciferase reporter assay and demonstrated that miR-29c could bind AKT2 (Fig. 4A). Then we overexpressed miR-29c in NSCLC cell lines SPC-A-1 and A549 by mimic transfection and detected the AKT2 protein level. In consequence, AKT2 showed obviously declining upon miR-29c overexpression comparing to negative control mimic transfection in both mRNA level and protein level in the two cell lines, indicating AKT2 is also a direct target of miR-29c in lung cancer (Fig. 4B). AKT1 and AKT2 are other two isoforms of AKT protein family. However, the binding site of miR-29c in AKT2 is unique which can be found neither in AKT1 nor AKT3. No change was found in protein level of AKT1 and AKT3 upon miR-29c overexpression (Fig. S3), which indicated that miR-29c had no regulatory effect on AKT1 and AKT3. Furthermore, the PI3K/AKT signaling pathway is related to the generation of chemotherapeutic resistance^25^. Based on these studies, we tried to elucidate the potential mechanism by which miR-29c decreases the cisplatin resistance of SPC-A-1 and A549 cells. We generated a hypothesis that there could be a link between miR-29c-induced cisplatin resistance and AKT2 expression. To confirm this, we used 10 µg/ml of cisplatin to treat SPC-A-1 and A549 cells for 12 hours. As a result, both the protein expression and mRNA expression of AKT2 were decreased post-treatment which showed the same effects to miR-29c overexpression. When we proceeded miR-29c overexpression and cisplatin treatment simultaneously, the AKT2 showed the lowest expression (Fig. 4C). Then, we overexpressed or knocked down the AKT2 expression using pcDNA3.1 vector or siRNAs, finding that the overexpression of AKT2 led to higher cell viability post cisplatin treatment compared with the control in both SPC-A-1 and A549 cells (Fig. 4D). In contrast, knocking down of AKT2 leads to the opposite results, where the si-AKT2 cells showed lower cell viability post cisplatin treatment, coincident with the miR-29c overexpression (Fig. 4D). When we performed “rescue” experiments by the overexpression of AKT2 with or without miR-29c, the AKT2 caused cisplatin resistance could be at least partly rescued by miR-29c overexpression (Fig. 4E). These results demonstrated that miR-29c could enhance the cisplatin sensitivity of SPC-A-1 and A549 cells by negatively regulating AKT2.

### MiR-29a and miR-29b have no significant regulatory effect on the regulation of lung cancer cisplatin resistance

**Figure 5:**
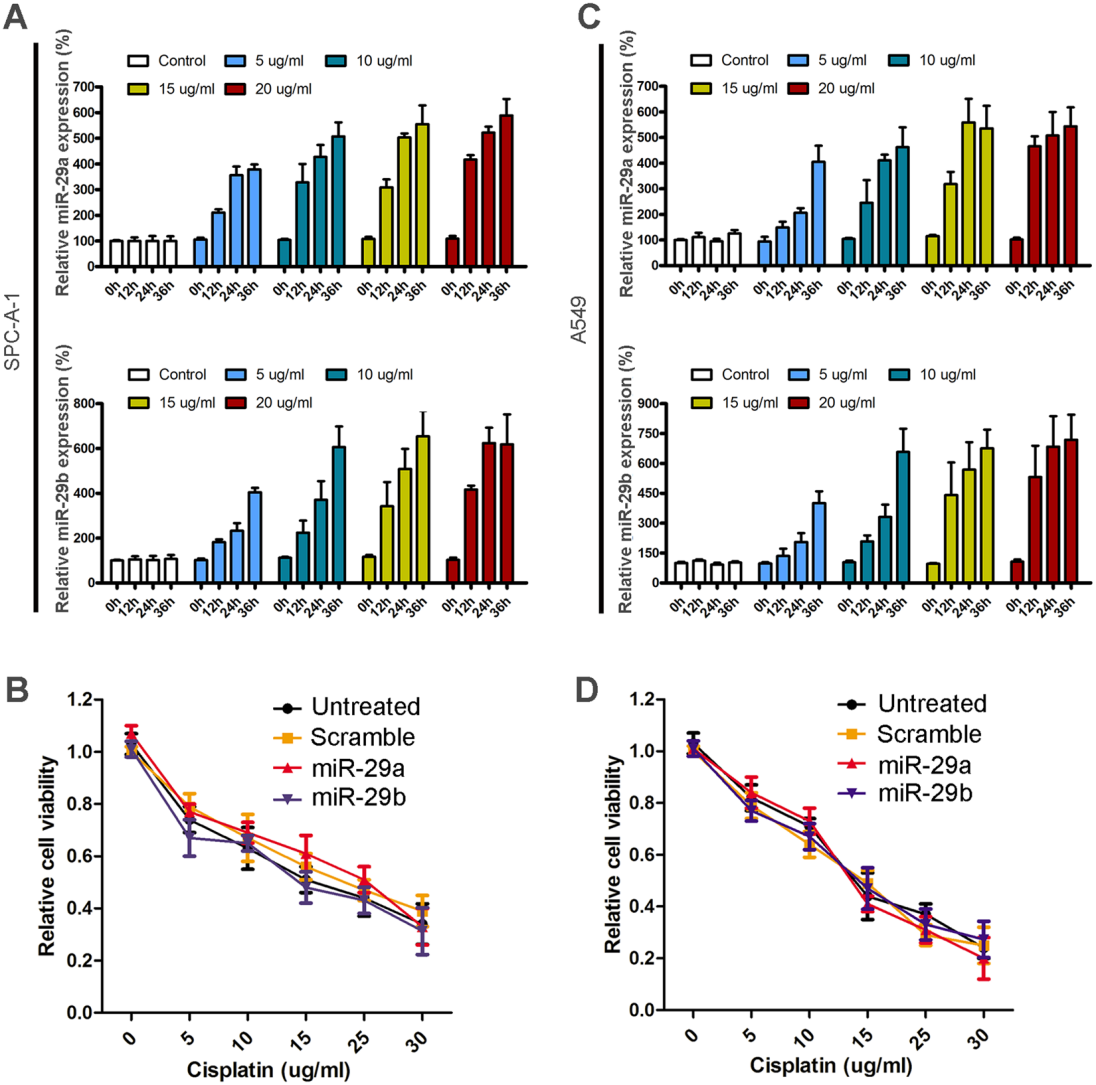
MiR-29a/b expression post cisplatin treatment and their effects on NSCLC cells cisplatin resistance. (**A**) and (**C**) Relative expression of miR-29a and miR-29b in SPC-A-1 and A549 cells post cisplatin treatment for 0, 12, 24 and 36 h. The expression of miR-29a or miR-29b in “control” group at 0 h was set as “100%” in SPC-A-1 cell (A) or A549 cell (**C**). (**B**) and (**D**) Effects of miR-29a and miR-29b overexpression on SPC-A-1 and A549 cell viability upon cisplatin treatment. To detect the relationship between miR-29a/b expression and NSCLC cell cisplatin resistance, 0, 5, 10, 15, or 20 µg/ml cisplatin was used to treat SPC-A-1 or A549 cells for 12 hours.

There are three members in miR-29s family, including miR-29a, miR-29b and miR-29c. Here we also detected the expression of miR-29a and miR-29b bu Real-time PCR in SPC-A-1 and A549 cells with or without cisplatin treatment. The potential function of miR-29a and miR-29b in regulating cisplatin sensitivity of SPC-A-1 and A549 was also detected by CCK8 assay. As a result, miR-29a and miR-29b are also upregulated by cisplatin treatment (Fig. 5A,C). However, when we overexpressed miR-29a and miR-29b by mimic transfection in SPC-A-1 and A549 cells, no significant regulatory effect was observed of the cell sensitivity upon cisplatin treatment (Fig. 5B,D), indicating that only miR-29c has negative regulatory roles during the cisplatin-resistant genesis of lung cancer cells.

## Discussion

MiRNAs play important roles during lung cancer tumorigenesis and development and can regulate cell proliferation, metastasis and chemotherapy drug sensitivity. During chemotherapy, cancer cells may exhibit abnormal gene expression, some of which has been demonstrated to take part in the regulation of cancer cell sensitivity to chemotherapy drugs.

Our study focused on the miRNA miR-29c, which was found to be downregulated in lung cancer tissue compared with adjacent normal tissue. Interestingly, clinic analyses suggested that there could be a correlation between miR-29c expression and the curative effect of the chemotherapeutic drug cisplatin. To examine this hypothesis, we used a concentration gradient of cisplatin to treat lung cancer cells, finding that cisplatin can enhance miR-29c expression. Then, a CCK8 assay was performed to measure the lung cancer cell viability upon cisplatin treatment in conditions of miR-29c overexpression or knockdown. We found that high expression of miR-29c led to cisplatin sensitivity, while low expression led to cisplatin resistance in lung cancer cells, demonstrating that there is likely a positive correlation between miR-29c expression and cisplatin sensitivity in lung cancer cells. In fact, miR-29c levels can be increased by chemotherapeutic drugs in some other cancers. For example, it can be upregulated by docetaxel, cisplatin, imatinib as well as celecoxib treatment in gastric cancer^26^.

The miR-29 family is a well-known gene in cancer, the tumor suppressor property of which has been identified in many cancers, including breast, gastric, ovarian and lung cancers^17,18,26–28^. There are three members in miR-29 family, including miR-29a, miR-29b and miR-29c. However, we did not observe any effect of miR-29a and miR-29b on the regulation of cisplatin sensitivity in SPC-A-1 and A549 cells. In gastric cancer, the expression of miR-29a, miR-29b and miR-29c can be activated by the chemotherapeutic drugs cisplatin or docetaxel. However, only miR-29c showed a clear relationship between expression and prognosis in patients with gastric cancer^26^. This phenomenon may suggest that miR-29c plays a much more important role in regulating cancer progression and chemotherapy resistance in gastric cancer. In ovarian cancer, the low expression of the miR-29 family is a cause for the genesis of cancer cell cisplatin resistance. All three members, miR-29a, miR-29b and miR-29c, play roles in this process^15^. In non-small cell lung cancer, miR-29b has been reported to function as a tumor suppressor gene, the lower expression of which tends to have shorter OS and RFS periods than their counterparts^28^. Here, we found that miR-29c expression was associated with NSCLC survival and cisplatin resistance. MiR-29a and miR-29b also showed a downregulation trend, but no significant difference was found between noncancerous adjacent tissues and NSCLC tissues. We failed to observe a correlation between miR-29a or miR-29b expression and NSCLC survival. This might be due to the limited number of patient samples and the varieties among clinical samples. Nevertheless, these two microRNAs could also play important roles via other regulatory mechanism. For incidence, miR-29a participate in DNA damage response through suppressing Cdc7 kinase in lung cancer cells^29^.

It is clear that the PI3K/AKT pathway plays important roles in regulating physiological and pathological processes. During tumorigenesis and progression, the PI3K/AKT pathway regulates multiple cell behaviors, including proliferation, migration and metastasis and so on. In addition, the PI3K/AKT pathway was demonstrated to function as a crucial pathway in the regulation of chemotherapy resistance generation of cancer cells^30,31^. AKT2 is a key molecule in the PI3K/AKT pathway, the aberrant expression of which can directly affect the function of the PI3K/AKT pathway. It has been confirmed that in gastric cancer, miR-29s can function as tumor suppressor gene via targeting and negatively regulating AKT2^18^. Here, we not only demonstrate the suppression of AKT2 is the route by which miR-29c functions as a tumor suppressor gene but also confirmed it is the route by which miR-29c regulates lung cancer cell cisplatin resistance genesis. However, it can be inferred from previous research that the function of the miR-29 family could be different among different cancer cell types. Therefore, whether or not miR-29c plays a role in chemotherapy sensitivity regulation in other cancer types needs further research.

Previous reports have assessed multiple function of miR-29s in lung cancer. For example, miR-29s suppress the Wnt signaling pathway through demethylation of WIF-1 in NSCLC^32^. While Muller *et al*. demonstrated that miR-29s modified DNA methylation status during lung cancer tumorigenesis by targeting DNA methyltransferases 3A and 3B, which were two key enzymes involved in DNA methylation^16^. These reports showed that miR-29s could take part in DNA methylation regulatory pathways, indicating miR-29s could function as multiple functional genes.

In summary, this study reports that miR-29c was frequently downregulated in NSCLC and acts as a tumor suppressor gene in NSCLC cells by negatively regulating AKT2. The upregulation of miR-29c enhances the sensitivity of SPC-A-1 and A549 cells to cisplatin. These results may explain the role of miR-29c in cisplatin chemoresistance and provide insight into the rational development of novel combinations of targeted drugs against NSCLC.

## Methods

### Patients and tissue samples

Pairs of matched NSCLC and noncancerous tissue samples were collected from 130 NSCLC patients (71 males and 59 females) who underwent surgical resection at the Shandong Cancer Hospital Affiliated to Shandong University between May 2011 and October 2015. We collected the tissue samples on condition of receiving the approval of Clinical Research Ethics Committee of Institute of Basic Medical Sciences, Chinese Academy of Medical Sciences as well as obtaining written informed consent from all participants. All methods were performed in accordance with the relevant guidelines and regulations of Clinical Research Ethics Committee of Institute of Basic Medical Sciences, Chinese Academy of Medical Sciences. After surgical resection, tissue samples were immediately frozen and stored in liquid nitrogen until RNA or protein extraction.

### Cell culture and transfection

Human NSCLC cell lines (SPC-A-1 and A549) were purchased from the Cancer Institute of the Chinese Academy of Medical Sciences. RPMI-1640 or Dulbecco’s modified Eagle’s medium supplemented with 100 U/ml penicillin, 100 U/ml streptomycin and 10% fetal bovine serum was used for the culture of SPC-A-1 and A549 cell lines at 37 °C in 5% CO_2_.

GenePharma (GenePharma, Shanghai, China) synthesized miRNA mimics, antagomiR and the negative control. The cells were transfected using Lipo3000 reagent (Life, CA, USA) according to the manufacturer’s instructions with a final mimic or antagomiR concentration of 10 nM.

### Cisplatin treatment

Cisplatin was diluted to different concentrations in physiological saline and physiological saline was used as a negative control. To detect the expression level of miR-29s, 0, 5, 10, 15 or 20 µg/ml of cisplatin was used to treat SPC-A-1 or A549 cells for 0, 12, 24 and 36 hours. To detect the relationship between miR-29s and NSCLC cell cisplatin resistance, 0, 5, 10, 15, or 20 µg/ml of cisplatin was used to treat SPC-A-1 or A549 cells for 12 hours. Afterwards, CCK8 was used to determine the cell survival rate.

### RNA extraction, cDNA synthesis, and real-time PCR assays

Trizol reagent (Life, CA, USA) was used to extract total RNA according to the standard manufacturer’s instructions. After extraction, RNA was quantified by absorbance at 260 and 280 nm and M-MLV reverse transcriptase (Invitrogen, CA, USA) was used for single strand cDNA synthesis. Oligo (dT)_18_ RT primers and a stem loop RT primer were respectively used for the reverse transcription of mRNA and miRNAs. Using a SYBR mix kit (Transgene, Beijing, China), quantitative real-time PCR (qRT-PCR) was carried out under the following conditions: 95 °C for 30 s, followed by 40 cycles of 95 °C for 5 s and 60 °C for 34 s. GAPDH was used as an endogenous control for normalizing mRNA and U6 snRNA was used as the endogenous control for miRNAs. Primer sequences used for qRT-PCR are listed in Table 1. The relative expression amounts were measured using the 2^−ΔΔCT^ method.

**Table 1:** Primers for real-time PCR.

Primer Name	Sequence (5′-3′)	Used for
miR-29a-F	TGCGCTAGCACATCTGAAAT	qRT-PCR
MiR-29b-F	CTGGAGTAGCACCATTTGAAAT	qRT-PCR
MiR-29c-F	CTGGAGTAGCACCATTTGAAAT	qRT-PCR
MiR-anti	GTGCAGGGTCCGAGGT	qRT-PCR
U6-F	CTCGCTTCGGCAGCACATATACT	qRT-PCR
U6-R	ACGCTTCACGAATTTGCGTGTC	qRT-PCR
AKT2-F	GCCCAGTCCATCACAATCAC	qRT-PCR
AKT2-R	GATGCTGGCCGAGTAGGAG	qRT-PCR
GAPDH-F	TCAACGACCACTTTGTCAAGCTCAGCT	qRT-PCR
GAPDH-R	GGTGGTCCAGGGGTCTTACT	qRT-PCR
Oligo dT	TTTTTTTTTTTTTTTTTT	Reverse transcription
U6-RT	AAAATATGGAACGCTTCACGAATTTG	Reverse transcription
29a-RT	GTCGTATCCAGTGCAGGGTCCGAGGTATTCGCACTGGATACGACTAACCG	Reverse transcription
29b-RT	GTCGTATCCAGTGCAGGGTCCGAGGTATTCGCACTGGATACGACAACACTG	Reverse transcription
29c-RT	GTCGTATCCAGTGCAGGGTCCGAGGTATTCGCACTGGATACGACTAACCG	Reverse transcription

### Cell survival rate assay

The mimic-miR-29s transfected cells or negative control transfected cells were seeded into 96-well plates (1000 cells/well). Cells in each well were incubated with 10 µl of CCK-8 (DOJINDO, Japan) diluted in normal culture medium at 37 °C for 2 hours. Survival rates were determined at 0, 12, 24, 48, 72, and 96 hours after transfection.

### Western blotting

A total of 30 µg of total protein was separated on a 12% SDS-PAGE gel, and then was transferred to PVDF membranes (Amersham, Buckinghamshire, UK). Membranes were blocked for 2 hours with 5% non-fat dried milk at room temperature and incubated overnight with an anti-AKT2 antibody (Bioworld, USA) at a 1:1500 dilution; the anti FHIT antibody (CST, USA) was used at 1:1000 dilution; the anti-GAPDH antibody (Proteintech, USA) was used at a 1: 40,000 dilution. After washing with TBST buffer (0.1% Tween-20, 150 mM NaCl, and 10 mM Tris, pH 8.0), the membranes were incubated for 2 h with a goat anti-rabbit antibody (Proteintech, USA) at either a 1:3000 or 1:30000 dilutions.

Histology experiments were performed using histology experiments staining kit from ZhongshanJinqiao biotechnology company (ZhongshanJinqiao, China). Slides were incubate overnight with an anti-AKT2 antibody (Bioworld, USA) at a 1:200 dilution; or the anti Ki-67 antibody (CST, USA) was used at 1:100 dilution.

### *In vivo* studies

The IACUC committee of Shandong University approved all animal experiments. Animal maintenance and experimental procedures were performed following strict adherence to the guidelines of the Animal Care and Use Committee. A total of 1 × 10^6^ A549 cells in 80 μl of FBS free medium were subcutaneously transplanted into 5-week-old female nude mice for each group (5 mice per group). When the average tumor size reached approximately 0.1 cm^3^, cisplatin was administered via intraperitoneal injection at a dose of 2 mg/kg at day 12, 18 and 24. Saline was used as a control. Tumor volumes were calculated based on the equation: V = (length × width^2^)/2. All mice were sacrificed at the 32^nd^ day and the tumor masses were weighted.

### Statistics

Each experiment was repeated at least three times. A two-tailed student’s t-test was performed. *indicates *p* < 0.05; **indicates *p* < 0.01; ***indicates *p* < 0.001; The mean ± SD is displayed in the figures.

## Electronic supplementary material

Supplementary information

## References

[1] Jemal A, Siegel R, Xu J, Ward E (2010). Cancer statistics, 2010. CA Cancer J Clin..

[2] Tomari Y, Zamore PD (2005). Perspective: machines for RNAi. Genes Dev..

[3] Engels BM, Hutvagner G (2006). Principles and effects of microRNA-mediated post-transcriptional gene regulation. Oncogene..

[4] Ambs S (2008). Genomic profiling of microRNA and messenger RNA reveals deregulated microRNA expression in prostate cancer. Cancer Res..

[5] Slack FJ, Weidhaas JB (2008). MicroRNA in cancer prognosis. N Engl J Med..

[6] Garzon R, Calin GA, Croce CM (2009). MicroRNAs in Cancer. Annu Rev Med..

[7] Guttilla IK, White BA (2009). Coordinate regulation of FOXO1 by miR-27a, miR-96, and miR-182 in breast cancer cells. J Biol Chem..

[8] Shuangni Yu (2010). miRNA-96 Suppresses KRAS and Functions as a Tumor Suppressor Gene in Pancreatic Cancer. Cancer Research..

[9] Menghini R (2009). MicroRNA 217 modulates endothelial cell senescence via silent information regulator 1. Circulation..

[10] Wu-Gan Zhao S-NY, Lu Z-H, Ma Y-H, Gu Y-M, Jie C (2010). The miR-217 microRNA functions as a potential tumor suppressor in pancreatic ductal adenocarcinoma by targeting KRAS. Carcinogenesis..

[11] Kato M (2009). TGF-beta activates Akt kinase through a microRNA-dependent amplifying circuit targeting PTEN. Nat Cell Biol..

[12] Kong G (2011). Upregulated microRNA-29a by hepatitis B virus X protein enhances hepatoma cell migration by targeting PTEN in cell culture model. PloS one..

[13] Gebeshuber CA, Zatloukal K, Martinez J (2009). miR-29a suppresses tristetraprolin, which is a regulator of epithelial polarity and metastasis. EMBO Rep..

[14] Xiong Y (2010). Effects of microRNA-29 on apoptosis, tumorigenicity, and prognosis of hepatocellular carcinoma. Hepatology..

[15] Yu PN (2014). Downregulation of miR-29 contributes to cisplatin resistance of ovarian cancer cells. Int J Cancer..

[16] Fabbri M (2007). MicroRNA-29 family reverts aberrant methylation in lung cancer by targeting DNA methyltransferases 3A and 3B. Proceedings of the National Academy of Sciences of the United States of America..

[17] Rothschild SI (2012). MicroRNA-29b is involved in the Src-ID1 signaling pathway and is dysregulated in human lung adenocarcinoma. Oncogene..

[18] Gong JA (2014). Characterization of microRNA-29 family expression and investigation of their mechanistic roles in gastric cancer. Carcinogenesis..

[19] Wu DW (2015). c-Myc suppresses microRNA-29b to promote tumor aggressiveness and poor outcomes in non-small cell lung cancer by targeting FHIT. Oncogene..

[20] Ma J, Dong C, Ji C (2010). MicroRNA and drug resistance. Cancer Gene Ther..

[21] Yu ZW (2010). MicroRNAs contribute to the chemoresistance of cisplatin in tongue squamous cell carcinoma lines. Oral Oncol..

[22] Sorrentino A (2008). Role of microRNAs in drug-resistant ovarian cancer cells. Gynecol Oncol..

[23] Bian HB, Pan X, Yang JS, Wang ZX, De W (2011). Upregulation of microRNA-451 increases cisplatin sensitivity of non-small cell lung cancer cell line (A549). J Exp Clin Cancer Res..

[24] Zhang H (2015). MicroRNA-29s could target AKT2 to inhibit gastric cancer cells invasion ability. Med Oncol..

[25] Lee S, C. E, Jin CB, Kim DH (2005). Activation of PI3K/Akt pathway by PTEN reduction and PIK3CA mRNA amplification contributes to cisplatin resistance in an ovarian cancer cell line. Gynecol Oncol..

[26] Wang Y (2015). Chemotherapy-Induced miRNA-29c/Catenin-delta Signaling Suppresses Metastasis in Gastric Cancer. Cancer Res..

[27] Yu PN (2013). Downregulation of miR-29 contributes to cisplatin resistance of ovarian cancer cells. Int J Cancer..

[28] Wu D-W (2015). c-Myc suppresses microRNA-29b to promote tumor aggressiveness and poor outcomes in non-small cell lung cancer by targeting FHIT. Oncogene..

[29] Barkley L. R., Santocanale C. MicroRNA-29a regulates the benzo[a]pyrene dihydrodiol epoxide-induced DNA damage response through Cdc7 kinase in lung cancer cells. 10.1038/oncsis.2013.20 PMC3740286 23877787

[30] Yu HG (2008). Phosphoinositide 3-kinase/Akt pathway plays an important role in chemoresistance of gastric cancer cells against etoposide and doxorubicin induced cell death. Int J Cancer..

[31] Lee S, Choi EJ, Jin C, Kim DH (2005). Activation of PI3K/Akt pathway by PTEN reduction and PIK3CA mRNA amplification contributes to cisplatin resistance in an ovarian cancer cell line. Gynecol Oncol..

[32] Tan M, Wu J, Cai Y (2013). Suppression of Wnt signaling by the miR-29 family is mediated by demethylation of WIF-1 in non-small-cell lung cancer. Biochem Biophys Res Commun..

