# Supplementary material for: MiR-29c reduces the cisplatin resistance of non-small cell lung cancer cells by negatively regulating the PI3K/Akt pathway
Source: Sci Rep. 2018 May 22;8:8007. doi: 10.1038/s41598-018-26381-w (PMC5964122; doi:10.1038/s41598-018-26381-w)
Supplement: Supplementary file 1 — Supplementary information [file 41598_2018_26381_MOESM1_ESM.pdf]

MiR-29c reduces the cisplatin resistance of non-small cell lung cancer cells by negatively regulating the PI3K/Akt pathway

Dian-min Sun*[1#], Bu-fu Tang[2#], Zhen-xiang Li[3], Hong-bo Guo[1], Jin-ling Cheng[1], Ping-ping Song[4], Xin Zhao[1]

*Corresponding author: Dian-min Sun, Department of Thoracic Surgery, Shandong Cancer Hospital affiliated to Shandong University, Shandong Academy of Medical Sciences. Tel.: +86 0531 67626232. Email: sun_dianmin@outlook.com

1: Department of Thoracic Surgery, Shandong Cancer Hospital Affiliated to Shandong University.

2: Department of Oncology, the First Affiliated Hospital of Dalian Medical University, Dalian, Liaoning Province.

3. Department of Radiation Oncology, Shandong Cancer Hospital Affiliated to Shandong University.

4: Department of Internal Medicine, Shandong Provincial Hospital affiliated to Shandong University.

#: The authors Dian-min Sun and Bu-fu Tang share co-first author to this work.

Supplementary figures

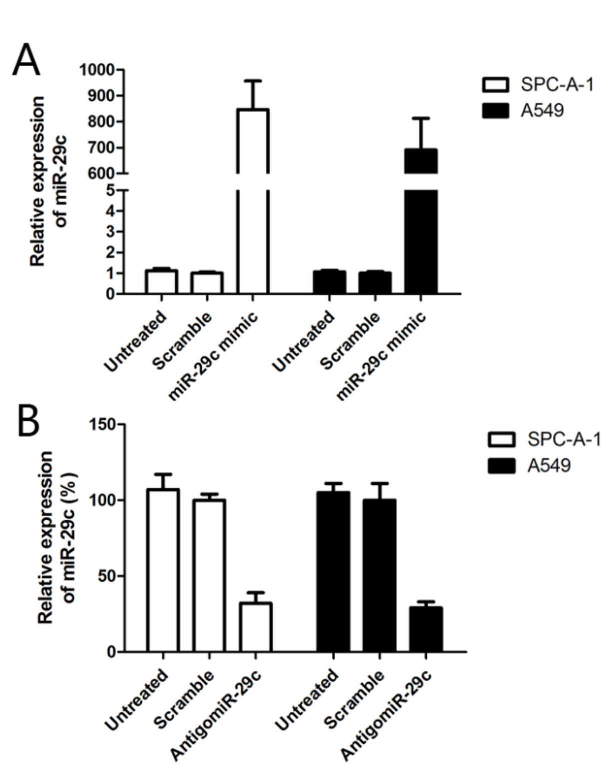

Figure S1. MiR-29c overexpression (A) or knock down (B) efficiency in SPC-A-1 and A549 cells. In A, the expression of miR-29c in "Scramble" group was set as "1". In B, the expression of miR-29c in "Scramble" group was set as "100%".

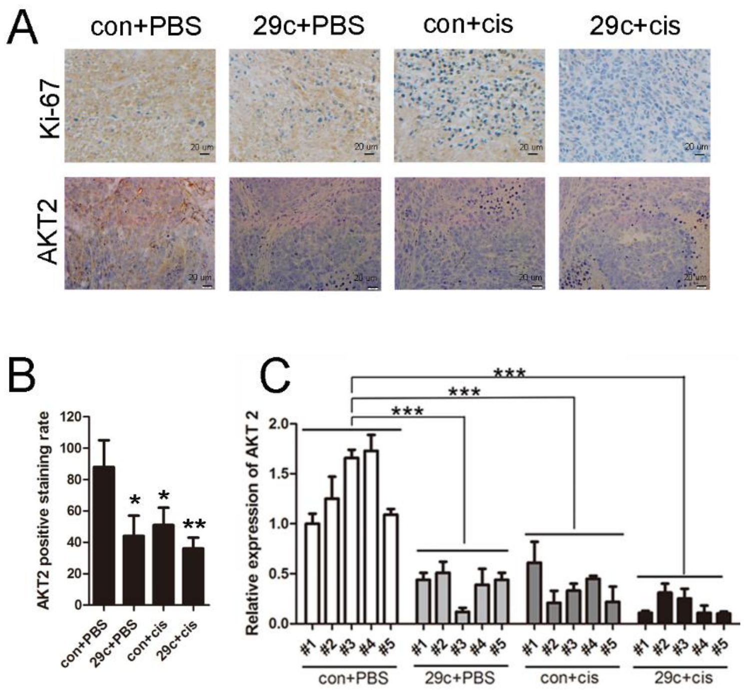

Figure S2. Ki-67 and AKT2 expression in mice tumors. A. Representative immunohistochemical staining of Ki-67 (up) and AKT2 (down) in each group. **B.** AKT2 positive rate in each group. **C.** The expression of AKT2 in mice tumors. Data was normalized to AKT2 expression of mice #1 in group “con+PBS”. “con” represents mimic control; “cis” represents “cisplatin”; “29c” represents “mimic miR-29c”.

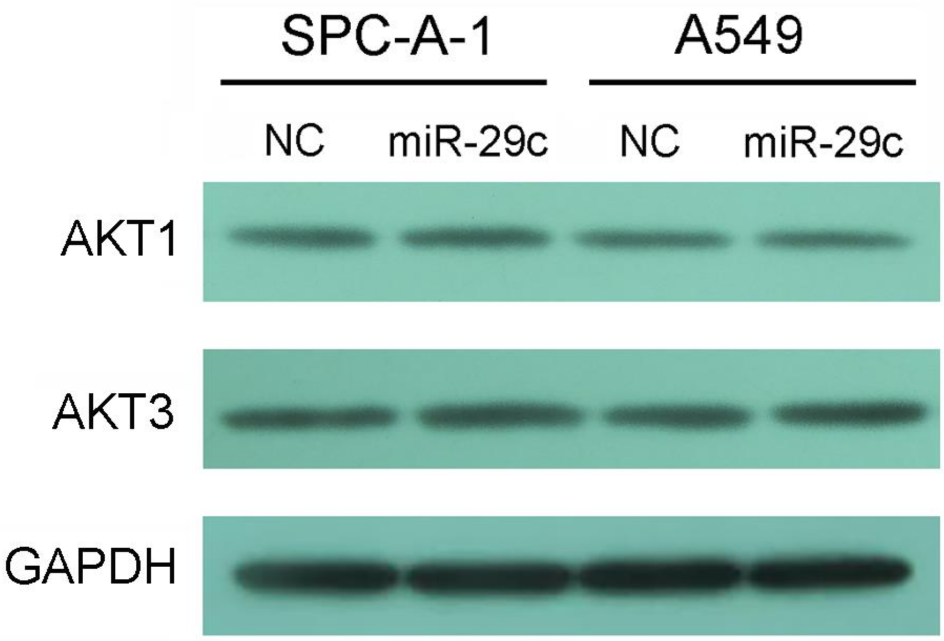

Figure S3. AKT1 and AKT3 protein expression with or without miR-29c over expression in SPC-A-1 and A549 cells. No significant difference was found between mimic control transfection and miR-29c transfection.
